# Blood-Based Biomarkers for Managing Workload in Athletes: Considerations and Recommendations for Evidence-Based Use of Established Biomarkers

**DOI:** 10.1007/s40279-023-01836-x

**Published:** 2023-05-19

**Authors:** Nils Haller, Michael Behringer, Thomas Reichel, Patrick Wahl, Perikles Simon, Karsten Krüger, Philipp Zimmer, Thomas Stöggl

**Affiliations:** 1 Department of Sports Medicine, Rehabilitation and Disease Prevention, Johannes Gutenberg University of Mainz, Mainz, Germany; 2 Department of Sport and Exercise Science, University of Salzburg, Schlossallee 49, Salzburg, 5400 Hallein-Rif Austria; 3 Department of Sports Sciences, Goethe University Frankfurt, Frankfurt am Main, Germany; 4 Department of Exercise Physiology and Sports Therapy, Institute of Sports Science, Justus-Liebig-University Gießen, Gießen, Germany; 5 Department of Exercise Physiology, German Sport University Cologne, Cologne, Germany; 6 Division of Performance and Health (Sports Medicine), Institute for Sport and Sport Science, TU Dortmund University, Dortmund, Germany; 7 Red Bull Athlete Performance Center, Salzburg, Austria

## Abstract

Blood-based biomarkers can provide an objective individualized measure of training load, recovery, and health status in order to reduce injury risk and maximize performance. Despite enormous potentials, especially owing to currently evolving technology, such as point-of-care testing, and advantages, in terms of objectivity and non-interference with the training process, there are several pitfalls in the use and interpretation of biomarkers. Confounding variables such as preanalytical conditions, inter-individual differences, or an individual chronic workload can lead to variance in resting levels. In addition, statistical considerations such as the detection of meaningful minimal changes are often neglected. The lack of generally applicable and individual reference levels further complicates the interpretation of level changes and thus load management via biomarkers. Here, the potentials and pitfalls of blood-based biomarkers are described, followed by an overview of established biomarkers currently used to support workload management. Creatine kinase is discussed in terms of its evidence for workload management to illustrate the limited applicability of established markers for workload management to date. We conclude with recommendations for best practices in the use and interpretation of biomarkers in a sport-specific context.

## Key Points

**Table:** 

Evidence-based use of established blood-based biomarkers requires consideration of the rationale, appropriate time points for measurement, and appropriate statistical analysis.
Even with widely used biomarkers (creatine kinase, urea, C-reactive protein, myoglobin, white blood cells), there are some pitfalls regarding their correct interpretation in the context of exercise. The example of creatine kinase is used to explain in detail which influencing factors (such as the release and clearance mechanism, measurement method, inter-individual differences) eventually affect the measured creatine kinase activity.
Biomarkers are suggested to be used with complementary objective and subjective monitoring tools such as questionnaires and performance data.

## Introduction

In recent years, elite sports have experienced an enormous surge in professionalization and commercialization. As a result, clubs, teams, and athletes face new challenges and opportunities. Concomitantly, the demands on athletes have increased owing to the evolving nature of sports and congested competition schedules. For instance, soccer has undergone a significant evolution in terms of the nature of the game (e.g., increase in running distance, number of runs, number of sprints, high-speed actions) as well as the number of games played during the season, resulting in elite athletes playing up to 75 games per season [1, 2]. Likewise, handball has developed into a faster game in terms of playing style because of rule innovations such as the introduction of “fast center” [3]. Furthermore, the COVID-19 pandemic further exacerbated the trend of congested schedules, as exemplified by the NBA schedule, where a game was played approximately every other day in the 2020/2021 season because of the delayed season start [4]. These circumstances present an increasing difficulty not only for the players but also for the practitioners and therefore require increased attention to monitoring training and competition load (i.e., the cumulative amount of stress placed on an individual from training and competition [5]) to assess and maximize training efficacy as well as manage or adjust the upcoming training and competition loads [6, 7].

On the one hand, the training load can be manipulated to elicit a desired training response (e.g., a stimulus that leads to positive adaptation) [8]. On the other hand, poor load management, i.e., prescribing, monitoring, and adjusting workload [5], is one of the contributing factors to non-functional overreaching (i.e., intensified training leading to performance stagnation [9]): illness, and injury. [5, 10–12]. In recent decades, several assessments have been suggested to facilitate decision-making processes for coaches. A plethora of these assessments refer to (i) acute load and recovery management and (ii) general resilience, i.e., the ability to cope with stressors (e.g., training load, psychological load, nutritional aspects such as food intolerance; in line with [10, 13–15]).

In order to monitor load holistically, both the external (“the physical work prescribed” [7]) and internal load (“the psychophysiological response” [7, 8]) should be assessed [16]. The external load can be expressed through, for example, time-motion analysis, tracking devices, or power meters (and through variables such as distance covered or peak power output). To determine the internal load, practitioners often rely on questionnaires such as the rate of perceived exertion (RPE) or heart rate (HR) responses during exercise [17, 18]. Recovery needs related to the training load are typically assessed with questionnaires such as the Acute Recovery and Stress Scale, neuromuscular performance tests, or aspects of HR such as HR variability [18–20].

While questionnaires are poorly objective and can be biased by, for example, social desirability (especially in team sports), or over- or underestimation of training load [21], performance tests might cause additional fatigue and interrupt the training routine. Heart rate has been shown to be a relevant single measure of internal load in endurance sports, but not in intermittent sports or resistance training [7, 8]. In addition, the diurnal variation in HR can complicate its interpretation [17, 22]. Jump performance is often considered a marker of neuromuscular fatigue, reflecting aspects of the acute and chronic load [20]. However, studies show conflicting results regarding its validity in different sports settings, with little knowledge about the relevant variables that should be analyzed [23].

To overcome these shortcomings, blood-based biomarkers (i.e. “indicators of normal biological processes, pathogenic processes, or responses to an exposure” [24]) such as lactate, urea, myoglobin, or creatine kinase (CK) are already routinely used in many areas of elite sport to objectively either determine the acute internal load or to estimate the internal load by assessing tissue- or organ-specific fatigue/stress/damage or recovery processes. In this way, they can contribute to a holistic picture of an athlete’s training response or condition and help coaches to individualize workload and prevent injuries [17, 22]. Through minimal capillary blood sampling, some already established biomarkers are repeatedly measurable and do not interfere with daily training routines. However, unlike clinical assessments, measurements and their interpretation are often based on personal experience rather than scientific evidence or relevance. In addition, before biomarkers can be used to facilitate decision making in practice, requirements such as standardized collection, selection of the appropriate markers (meeting criteria such as specificity and sensitivity), and repeated sampling to determine individual reference ranges should be met [25–27]. In addition to capturing aspects of load and recovery, another important dimension of blood collection is monitoring the health status of athletes, which in turn may also affect training and recovery prescriptions. Hematological parameters may inform practitioners on impeding or acute diseases. In addition, information on the iron balance of athletes can be obtained with blood sampling. For example, in female soccer players, iron deficiency anemia has been found in approximately 30% of athletes, even at the highest level [28], directly affecting maximal performance and underscoring the importance of monitoring aspects of athlete health through selected blood-based biomarkers.

In contrast to monitoring load, recovery, and health, blood-based biomarkers are to date rather rarely used to assess aspects of resilience. In the field of professional sports, many providers offer clubs and coaches a special added value through point-of-care, genetic, and epigenetic testing. Vendors claim to determine scientifically relevant biomarkers or genetic sequences to assess resilience, injury risk, or even aspects of athletic talent [29, 30]. To the knowledge of this expert group, unfortunately, none of these markers is yet validated and evidence-based for such prognostic purposes in the context of professional sports.

Thus, to date, practitioners often rely on practical, scientifically well-researched, and rapidly measurable biomarkers such as CK or lactate. This review aims to highlight selected well-studied blood-based biomarkers used to assess load, fatigue, and recovery processes. Potentials and pitfalls in collecting and analyzing these markers are emphasized, and finally, recommendations for best practice are provided for practitioners on how to use and interpret them in a sport-specific context.

## Biomarkers and the Potential for Objective Athlete Monitoring

**Fig. 1:**
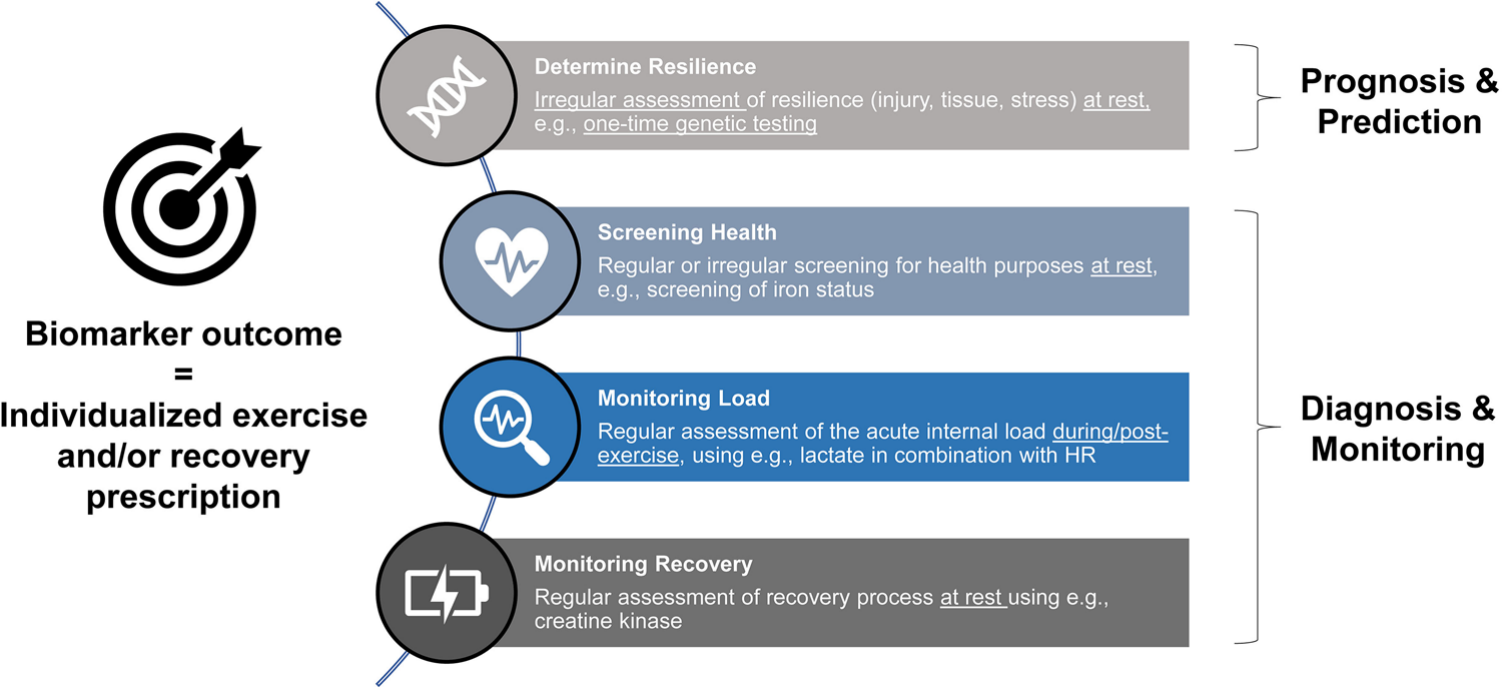
Different rationales for biomarker sampling: from resilience assessments, regular or irregular health monitoring, to daily monitoring of load and recovery/. *HR* heart rate

In general, blood biomarkers have great potential for the objective assessment of load, recovery, and general health if sensitive and context-specific markers are selected [31]. Biomarkers are applied for a variety of reasons, including prognosis, or diagnosis of fatigue or injury as well as disease or nutritional deficiencies [32]. There is a large amount of markers that are potentially capable of reflecting physiological alterations such as muscle damage, or inflammatory responses that typically occur after strenuous exercise. The idea of taking blood markers (or a blood marker panel) to map these alterations and providing individualized training recommendations based on these alterations to optimize performance and prevent injuries is appealing and can work under certain circumstances. Figure 1 outlines the different rationales for biomarker sampling.

There is consensus [9, 12] that markers used for monitoring purposes should be responsive and specific to training load, ideally unaffected by confounding factors such as diet or diurnal rhythm. They should be easily and frequently measurable. The result should be available without substantial delay and the measurement should not interfere with the training process. Many practically relevant blood parameters fulfill parts of these criteria. They can even be measured with a minimum of capillary blood, so that elaborate venous blood sampling is not necessary, which is often perceived as disruptive by athletes [27, 33]. The small amount of blood needed allows for repeated measurement without interfering with the training routine. Many biomarkers were shown to be sensitive in both acute and chronic exercise settings [34, 35]. Markers collected under resting conditions could detect the onset of overtraining (i.e., long-term decrement in performance capacity (…) in which restoration of performance capacity may take several weeks or months” [9]), but this has only been demonstrated in a few studies, for example, for cell-free DNA in a resistance training setting [36]. In this study, it should be noted that the definition of overtraining was not actually met, as there was no follow-up to confirm a long-term decline in performance. Correlations between changes in CK or high-sensitivity interleukin-6 levels and specific performance traits such as sprint distance during acute intermittent exercise indicate that biomarkers may also reflect aspects of the external exercise load [37, 38].

Note, however, that a correlation with an objective performance variable, such as sprint distance during exercise does not necessarily translate into a for example reflection of perceived fatigue or an increased injury risk. In this regard, Saw and colleagues [39] have shown that subjective well-being and biomarker levels are generally not well correlated, while questionnaires reliably respond to changes in training load. As outlined in the “BEST” resource [24] on biomarkers, a correlation with an objective performance variable does not automatically qualify a biomarker to be a validated surrogate endpoint (in the sports context for e.g., internal load, muscular fatigue, or injury risk). This is often assumed for established biomarkers, but there is a lack of rigorous analytical and clinical validation [40] of these biomarkers in their respective sports contexts. These considerations already highlight some of the challenges associated with the use of biomarkers for load management in athletes.

## Evidence and Usefulness of Currently Used Biomarkers for Workload Management

In recent years, much attention has been devoted to the development of novel biomarkers in the context of load monitoring with limited success to date. For many of these biomarkers, reference ranges do not exist, or their physiological backgrounds are unknown, and some require sophisticated measurement methods, making them difficult to use for monitoring purposes. Sports scientists, thus, typically rely on established markers such as blood lactate [41], CK, C-reactive protein (CRP), or urea to monitor load, recovery, and aspects of health. Table 1 outlines six practical biomarkers in this regard.

**Table 1:** Practical biomarkers with reference ranges, interpretation, and correlation with training load and questionnaire scores

System	Biomarker	Sampling site and medium	Confounding variables	Reference levels at rest	Interpretation of increased levels	Association with training load	Correlation with questionnaire scores	Reliability
Muscle	CK	Serum, capillary (ear lobe, fingertip) EDTA, or heparin plasma	Myocardial damage, mechanical impacts [42], glutamine intake [43], and circadian rhythm [44]	F: 30–135 U·L^−1^; M: 55–170 U·L^−1^ [45]; higher reference intervals for athletes (M: 82–1083 U·L^−1^; F: 47–513 U·L^−1^) [26]	Mechanical load, severe metabolic load [46]	Increases after acute exercise [38] peaking 1 to several days after the exercise depending on the type of exercise [47, 48]; mixed findings with respect to chronic training load throughout a season/training period; no changes [49–51], changes [52]	Mixed; from no relationship with DOMS [53] up to significant correlation with muscle soreness [54]	Good relative reproducibility (ICC = 0.9), poor absolute reproducibility (CV = 20%; [55])
Muscle	Myoglobin	Serum, capillary (ear lobe, fingertip) EDTA or heparin plasma	Myocardial damage, mechanical impacts [42] glutamine intake [43], and renal disease failure of clearance [56]	M: < 85 ng·mL^−1^ [57]	Mechanical load, severe metabolic load [58]	Rapidly increases after acute exercise [38]; rapid re-regulation towards baseline [59]; no chronic changes during a competitive American Football season [60]	Significant correlation (r = 0.73; *p* < 0.05) with increased Visual Analog Scale scores [61]	Good reproducibility (ICC = 0.75) [62]
Muscle	Lactate	Capillary (ear lobe, fingertip); EDTA whole blood or plasma	Nutritional aspects (especially CH) [63, 64]; sampling site [65, 66], treadmill parameters [67], and protocol [68]	0.3–1.5 mmol·L^−1^ [69]	High metabolic load (high energy demand), high glycolytic flux	Increases with increasing exercise intensity [70]; chronic decreases depend on the type of training [71]	Correlations up to r = 0.98 with RPE [72–74]	Good-to-excellent reproducibility during exercise (ICC = 0.75–0.99) [75]
Immune	WBC	EDTA whole blood	Any inflammatory condition, e.g., infections, injuries, and chronic inflammatory diseases	4–11 10^9^·L^−1^ [76, 77]	Infections, cellular inflammation	Increases with exercise intensity [78] and duration [79]; chronically decreased levels at rest and less pronounced increase in response to acute exercise [80]	Not associated [39]	Moderate reproducibility (ICC = 0.51–0.74) [35]
Metabolism	Urea	Serum, capillary (ear lobe, fingertip) EDTA, or heparin plasma	Nutrition (protein and fluid intake), sweating [81, 82], plasma volume, and lean body mass [83]	F: 4.0–6.0 mmol·L^−1^; M: 5.0–7.0 mmol·L^−1^ [81]; BUN 8–26 mg·dL^−1^ [84]	Enhanced protein catabolism [82]	Acute increases after triathlon [85]; no changes after a soccer game [86] or during/after a HIT shock cycle [87]; no chronic changes during a phase of overreaching [88] and a 6-week intensified cycling period [89]; increases during a simulated training camp [90]	One study reported correlations with each day’s intensity rating and next morning BUN levels [83]	N/A
Inflammation	CRP	Capillary (fingertip); serum, EDTA whole blood, or plasma	Inflammatory conditions, acute and chronic diseases [91–93], and diet [94]	< 10 mg·L^−1^ [95]	Exercise-induced inflammation, infectious or non-infectious diseases	Increases after acute exercise [96], peaking around 24 h post-exercise [35, 47]; no intensity dependent differences after 30-min running exercise [96]; chronic increases in a weightlifting setting over several weeks [36]; chronic decreases with increased level of regular physical activity [97]	In a study of handball players over 40 weeks, occasional correlations were found between subscales of the RESTQ-S questionnaire and CRP levels [49]	Moderate reproducibility (ICC = 0.71) [35]

*BUN* blood urea nitrogen, *CH* carbohydrate, *CK* creatine kinase, *CRP* C−reactive protein, *CV* coefficient of variation, *DOMS* delayed onset of muscle soreness, *EDTA* ethylenediamine tetraacetic acid, *f* female, *ICC* intra−class coefficient, *M* male, *N/A* not applicable, *RESTQ−S* Recovery Stress Questionnaire for Athletes, *WBC* white blood cells

Lactate is probably one of the most used and studied blood biomarkers in sports science. During exercise, lactate is manly produced and released by skeletal muscles (however, skeletal muscles also play an important role in lactate clearance) [98]. Muscle production of lactate is essential to remove pyruvate, regenerate NAD^+^ to sustain a high rate of ATP regeneration from glycolysis, and contribute to metabolic proton buffering [99]. On the one hand, lactate is valuable for determining intensity thresholds in physiological exercise testing [100]. On the other hand, it is also suitable for measuring acute exercise intensity (based on an individual lactate profile [101]). It has been shown that the combination of lactate and HR measures was well correlated with the RPE during high-intensity soccer exercise [102] but not during submaximal cycling exercise [103]. Lactate can be determined in venous blood, but most conveniently from the fingertips, toes, and earlobes [65, 66]. In this regard, one study has shown that lactate levels during submaximal rowing exercise are slightly but not significantly different between the earlobe, toe, and fingertip, suggesting toes as an equivalent alternative to earlobes and fingertips [66]. Feliu et al. [104]: however, demonstrated significant differences between earlobes and fingertips during graded exercise. Although all sampling sites are potentially suitable, these results indicate that practitioners should decide on one sampling site, while changing sampling sites during longitudinal monitoring should be avoided because of the potential differences between these methods; a principle that can also be applied to portable lactate systems from different manufacturers [105]. Although lactate can be considered a useful and load-sensitive variable in the acute exercise setting, there are variables that can affect the measured lactate levels, including lactate production, elimination, transportation, and blood flow [106]. Nutritional aspects such as low-carbohydrate availability may cause lactate thresholds to shift toward a higher workload on a short-term low-carbohydrate diet compared with a mixed diet [107]. Practitioners should be aware of such factors, especially during periods of repeated high-intensity training. While glycogen depletion may occur and lactate levels may be lower during subsequent training sessions, this should not necessarily be interpreted as an improvement in training status [108].

Urea, the excreted form of nitrogen in humans, is an end product of the degradation of nitrogenous or protein materials. Serum amino acids as well as proteins in serum and skeletal muscle are the most likely nitrogen sources. Historically, urea has been considered as a marker reflecting acute and chronic workloads, particularly in high-volume, high-intensity endurance sports, where maintaining a nitrogen balance is challenging because of high caloric expenditure [90, 109]. However, research over the past decades has shed light on the aspect that the measurement of urea in blood, without taking urinary excretion rates and losses of urea by sweating into account, does not seem to reflect load aspects sufficiently. As it is unrealistic to monitor both excretion rate in sweat and urine in other than basic science research projects, this may hinder the use of urea as a biomarker for load management in practice. In particular, neither a longitudinal study with repeated measurements in football [110], nor during and after an acute high-intensity training shock cycle [87], nor phases of overreaching (i.e., short-term decrement in performance capacity [9]) in rugby players [88] have revealed practical evidence that urea could be used for monitoring load in athletes. In addition, no acute changes of urea were detected after a soccer game [86]. However, one study highlighted the potential of urea to possibly reflect aspects of fatigue in cyclists during a simulated training camp [90] and a systematic review discussed the potential of urea to reflect changes in performance [111]. So far, there is thus conflicting evidence for urea reflecting aspects of athlete load and fatigue during acute exercise as well as periods of exceptionally high training volumes.

Myoglobin is a globular muscle protein found in cardiac and skeletal muscle cells. This muscle protein contains a heme group and is responsible for the intramuscular transport of oxygen by accepting the oxygen molecule from the hemoglobin of the blood before transporting it to the sites of oxidation. It is assumed that damage to muscle cells leads to a change in membrane permeability allowing the protein to enter the extracellular space. Because of its small molecular size of 17 kDa, myoglobin enters the circulation rapidly and is measurable in the blood as early as 1–2 h after a myocardial infarction. In addition, it disappears comparatively quickly (within 12–24 h) from the circulation owing to rapid renal clearance [112]. Because of its low specificity for cardiac muscle tissue, myoglobin no longer plays a significant role in cardiac diagnostics. In sports medicine, however, it is important as an indirect marker of damage to skeletal muscle because of its rapid kinetics. The literature shows that myoglobin increases after physical stress in sports. For example, Thorpe and Sunderland [38] reported a 238% increase of myoglobin after a soccer match in seven semi-professional soccer players and Fransson et al. [59] observed a seven-fold increase of myoglobin immediately after a 2 × 45-min simulated soccer game that returned to baseline within 24 h after recovery. As excessive excretion of myoglobin can lead to kidney damage and even kidney failure because of its toxic effect on renal tubules, the level of myoglobin in serum plays an additional important role for diagnostics in sports medicine [113]. Accordingly, myoglobin could be useful as a short-term muscle damage marker in acute settings. However, it is less evident whether this marker also responds to chronic load and whether it is suitable as a monitoring marker in seasonal cycles for sports practice [114]. Furthermore, it is unclear how to interpret myoglobin increases from the perspective of load management. Should increases be considered negative, or do they signal an intense yet effective training stimulus to some extent? If so, where is the limit? These questions are yet unresolved and should be considered when interpreting load-induced myoglobin increases. There is also ambiguity about the underlying mechanism of release, such as whether membrane damage actually occurs and whether an increase in blood myoglobin can be used as an indirect marker of such damage.

Intermediate increased numbers of circulating leukocytes, also known as white blood cells (WBCs), are an indicator for acute infections whereas chronic elevations serve as a prognostic marker for chronic diseases. Circulating WBCs make up less than 5% of the body’s total leukocyte pool. One should keep in mind that circulating levels of WBCs or their subsets do not necessarily reflect tissue-specific immunological reactions. In 1902, Ralph Larrabee was the first person describing elevated WBCs after the Boston Marathon [115, 116]. Today, it is well accepted that, similar to inflammatory humoral markers, WBCs increase during and after single bouts of exercise depending on exercise modality, intensity, and duration, with the most pronounced responses after long-term, intensive endurance exercise sessions [77–79, 85]. Fitness level, nutritional status, sex, age, and temperature may also influence both WBC resting levels and the acute exercise response. Evidence from preclinical and clinical studies suggest that the increase in WBCs in response to acute exercise is mainly driven by stress hormones, such as catecholamines and cortisol. It is accepted that this fast fight-or-flight reaction preconditions the human body for the contact with potential penetrating pathogens. It therefore makes sense that especially cells of the fast innate immune system are strongly affected (granulocytes, natural killer cells [77]). A more detailed analysis indicates that WBC subsets, such as lymphocytes (16–45%), granulocytes (45–75%), and monocytes (4–10%), respond differentially, especially in the hours after cessation of exercise. Circulating lymphocyte numbers and proportions, and especially the natural killer cell subset show a biphasic pattern. Lymphocytes increase immediately after cessation of exercise before they strongly decrease for up to 36 h after exercise, whereas T- and B-lymphocyte decreases are less pronounced and usually return to the baseline level within 6 h. During that second phase, lymphocyte counts may decrease 50% below resting levels. Although less pronounced, monocytes also increase after different types of exercise and return to baseline levels within 2 h post-exercise [77]. In contrast, granulocyte numbers, which account for approximately 66% of WBCs, further increase after the cessation of exercise for 4–6 h. Routinely taken blood pictures (hemograms) provide the opportunity to calculate more informative and integrative clinical markers, such as the neutrophil–lymphocyte-ratio, the platelet-lymphocyte-ratio, and the systemic inflammation index [117–120]. However, its applicability in the acute exercise setting is limited by the need for venous blood samples. Nevertheless, hemograms or more advanced methods such as a flow cytometry analysis may be used to inform practitioners on chronic inflammatory conditions (e.g., infections [121]). Resting levels of circulating WBCs should not differ from age- and sex-related reference values. More sophisticated assessments can provide further information on immune cell migration patterns and their function.

C-reactive protein, a marker of systemic inflammation, is an acute-phase protein produced mainly in the liver and has demonstrated clinical relevance owing to its increased levels under various pathological conditions such as autoimmune diseases, infections, or non-infectious diseases [91, 92] including prognostic relevance regarding cardiovascular events [93]. Interestingly, several studies have shown short-term increases after exercise. For example, one study showed about a 152-fold increase after running an ultradistance foot race that lasted more than 24 h [122]. A rather moderate acute increase was shown after a soccer game by Ispirlidis et al. [47] with levels peaking 24 h post-exercise. No immediate increase in CRP but 24 h post-exercise was shown in two further studies after both 60 min of intense endurance running [35] and eccentric cycling exercise [123]. Thirty minutes of endurance running at different intensities (65% vs 85% of VO_2max_) resulted in a roughly two-fold significant CRP increase post-exercise, but not in a significant intensity-related difference between both protocols [96]. While exhaustive resistance training over several weeks has led to chronic CRP increases [36], it was also shown that long-term physical activity leads to a decrease in resting levels probably through a decrease in cytokine production [97]. In addition, lifestyle modifications such as changes in diet may affect CRP resting levels [94]. Elevated resting levels can therefore be interpreted in two ways: first, it may signal an impending or acute illness, and second, it may indicate an inflammatory response triggered by exercise. Given that acute exercise below 24 h duration does not seem to provoke as massive a response as that observed 24 h after the cessation of a sport, CRP primarily shows a delayed type of response to exercise, like CK.

Taken together, these markers appear at first glance to be quite well suited for monitoring in selected sports contexts. In the following section, CK is discussed exemplarily from different points of view to illustrate problems of validity, interpretation of level changes, and physiological background. Some of these issues also apply to the biomarkers discussed in this section. The primary purpose of the following section is to point out that practitioners need to be cautious in their interpretations and that they also need to consider alternative explanations for biomarker increases and/or differences between athletes.

## What About the Evidence of Commonly Used Biomarkers in Sports Medicine: Using CK as an Example?

Creatine kinase, also known as creatine phosphokinase or phosphocreatine kinase, is an enzyme that catalyzes the reversible reaction of phosphocreatine, magnesium adenosine diphosphate (MgADP^−^), and a hydrogen ion to MgATP^2−^ and creatine to either re-phosphorylate ADP to ATP or store immediately available energy in the form of ATP in phosphocreatine [124]. Creatine kinase is frequently used in sports medicine as an indirect marker of muscle damage [26]. It is well known that the CK level in the blood commonly increases after unaccustomed exercises, especially when including eccentric contractions [125]. It is assumed that the mechanical stress placed on the muscle results in membrane damage [126], allowing the large CK molecule to leave the cell and to enter the bloodstream. Because of its molecular size of about 82 kDa, CK is unable to enter the bloodstream via the transepithelial pathway and must therefore be cleared from the interstitial fluid by the lymphatic vessels [127]. Its lymphatic transport is thought to be responsible for the delay from the damaging exercise and its detectability in the blood [128].

Although these are the hypothesized mechanisms underlying an exercise-induced CK increase, the pathophysiological background is much more complex and not yet understood in detail [129]. This fact is important to consider when interpreting CK levels and using this biomarker to guide exercise. First, the central assumption of exercise-induced cell membrane damage must be critically questioned. Although some animal studies found damage to the sarcolemma in 10–30% of muscle fibers after eccentric contractions as indicated by a lack of staining for membrane proteins [130], the proportion of damaged muscle fibers was considerably lower in human studies [131, 132] or even no significant damage to the sarcolemma could be detected [133]. Thus, we do not yet know exactly how CK leaves the muscle cell. Incidentally, the same applies to indirect damage markers in cardiac diagnostics. Here, high muscle protein levels are also sometimes found in the blood without significant membrane damage to the cardiomyocytes. Based on this paradox, Hickman et al. [134] proposed an alternative theory that the sarcolemma of cardiomyocytes under ischemic conditions forms membrane bubbles (blebs) filled with sarcoplasm that can detach (blebosome, microparticle) under sustained ischemia, allowing CK to leave the cell without detectable membrane damage. It has been speculated that under extreme metabolic stress, the skeletal muscle cells may act similarly, providing an explanation for high CK values after exhausting exercise without sarcolemmal damage [129, 135].

Second, lymphatic clearance of CK from the extracellular space leads to difficulties in interpreting enzyme activity in blood. This is partly owing to the fact that lymphatic transport from the damaged tissue to the venous angle of the left subclavian and internal jugular veins takes time, resulting in a delay between the release from the muscles and measurability in the blood. Therefore, not only the start and the end of the release can only be measured with a delay, but also all activities that influence the lymph flow will change the results. This is supported by the data of Havas et al. [136] who reported that a 23-h bedrest after a 18-km cross-country run resulted in a delayed and diminished CK response in the blood when compared with habitual activity. Similarly, Schillinger et al. [137] could show that lymphatic drainage following a treadmill exercise affected the CK response in terms of an accelerated decrease of the serum enzyme activity. However, others did not find any effect of lymphatic drainage, rest, or local cryotherapy on the CK kinetic after eccentric contractions [138].

The next problem arises from the fact that we usually measure the enzyme’s catalytic activity, expressed in international units per liter (IU·L^−1^). Therefore, we only record the number of active enzymes, while ignoring already inactivated enzymes. If the proportion of inactivation varies intra- or inter-individually, this will result in varying degrees of underestimation of the actual muscle damage. This problem was already identified in the early 1990s in the diagnostics of myocardial infarctions and led to the recognition that CK-MB (i.e., the isoenzyme) mass determination is superior to determining enzyme activity, when used for infarct sizing [139]. In the context of exercise-induced muscle damage, some data indicate that the blunted CK response after a repeated bout of eccentric contractions is the consequence of an increased enzyme inactivation kinetic, as evidenced by an increased ratio of inactive to active CK molecules following the second bout [140]. Another fact that has been known for decades, but has hitherto been ignored in sports medicine, is the positive relationship between muscle mass and CK activity in the blood. Garcia [141] and later Novak and Tillery [142] pointed out in the 1970s that this is another pitfall to consider when interpreting CK values. In addition, ethnicity appears to have an impact on CK levels. For example, Brewster et al. [143] reported that black individuals have higher CK levels than South Asian individuals and white individuals, which may result from higher CK activity in those tissues with high and fluctuating energy demands [144] and a larger muscle mass in black individuals [145]. Another problem of determining the level of muscle proteins as indirect markers of damage in the blood is based on the fact that impact trauma (e.g., a hit or body check) can also drastically increase the level of these proteins without reflecting the internal stress of the muscles [42, 146].

In view of the unknown physiological background, the question inevitably arises about the validated surrogate endpoint (see Sect. 2) but also of the correct interpretation of the levels. It is often assumed that the CK response positively correlates with muscle damage. However, there is often a poor relationship between functional outcomes and CK activity [147]. In the clinical population, 3000–5000 U·L^−1^ are considered abnormal or pathological, i.e., possibly associated with an increased risk of acute kidney injury [148, 149] whereas levels greater than 3000 U·L^−1^ have been detected in serum after for example, maximal resistance exercise training [150]. Therefore, absolute levels can only be of limited value for certain outcomes or pathologies, especially as there seem to be “high responders” who could reach remarkably high levels more quickly [151]. Apart from the problem that commercially available instruments often only measure diluted samples in ranges above 1500 U·L [152], the interpretation of CK levels is not straightforward: is an exercise-induced increase in CK levels a sign of a necessary stimulus that triggers a positive adaptive response, a result of an impact, a sign of excessive exercise, or even an indication of a pathological condition? This is a widely ignored question that can probably only be answered on an individual basis and by taking a long-term view of an athlete.

Moreover, the question arises from the optimal time point to collect the blood sample after exercise as CK does not necessarily peak within minutes post-exercise but rather between 24 and 120 h depending on the exercise modality [48]. As the next training session/competition is often close at hand, it is possible that the peak level is not reached before training is resumed. Confounding factors such as ethnicity, body composition, or exercising with different individual intensities can lead to highly individual kinetics and time points of peak CK levels [144, 153]. This complicates the question of the “right” time to collect blood, although it is also unclear whether the peak should be the relevant parameter at all.

Finally, most studies conducted in this area were observational in nature, i.e., researchers examined CK responses to exercise in both acute and chronic situations [37, 154–156]. In this regard, CK levels were occasionally shown to be correlated with variables such as sprint distance [38] or high-speed running [157] and even related to different positions in soccer [154] at rest. However, conclusions that “CK can be used to individualize load or recovery” [154] are somewhat problematic. To our knowledge, there are hardly any studies to support this idea. Sperlich et al. [158] showed in an innovative study design that workload management, i.e., monitoring and adjusting training load, using CK, among other variables, is possible in principle. The investigators intervened when physiological parameters were above reference values measured at baseline, and they reported no adverse events and an increase in performance using this approach. However, there is no evidence that the outcome was actually affected by changes made in the training load. This illustrates a major problem in the athlete population in general: large controlled studies that are needed to draw these conclusions are difficult to implement in elite athlete cohorts.

## Practical Applications for Practitioners in Elite Sport Settings

Biomarkers are used to obtain objective information on load and recovery, but even for an established biomarker there is limited evidence that CK is “fit for purpose” [24] for its use in load management (see Sect. 4). How can practitioners still add value?

### Preliminary Considerations

A clear rationale for blood collection from athletes should be established in advance to determine the appropriate timing and frequency of sample collection [31]. For instance, does a practitioner want to assess the health status or monitor the training load on a daily basis (see Fig. 1)? Health screening through hematological or immunological examinations in athletes aims less at monitoring the acute load than at identifying deficiencies or risks of disease, which can then be counteracted, for example, by recommending nutritional supplements [27, 31, 159]. A defined periodic measurement at rest (e.g., once per month) or in the event of a particular suspicion (e.g., when an athlete is underperforming) addresses this issue. In contrast, load-sensitive biomarkers such as lactate measured before, during, and after exercise reflect aspects of the acute psychophysiological response [8], which can be an objective tool to assess the efficacy of a training session [102]. Damage markers such as CK, measured regularly at rest on the days following exercise, indirectly reflect the degree of tissue-specific damage (and rather estimates the internal load retrospectively) and parts of the recovery process or the readiness for a new load. Regardless of the reason for blood sampling, the results should at least serve as a basis for decision making, otherwise, a considerable effort will remain without additional benefit.

Unlike questionnaires, blood collection and the timely processing to obtain prompt results (immediately before or after a given load, or both) are logistical challenges, especially for a team with many athletes. Blood collection must not interfere with the training or put too much load on athletes (especially on young athletes) in terms of collection frequency and blood volume. In this regard, coach/staff buy-in is essential for the implementation, but also for the sustainability of such approaches. Practitioners need to see an added value that increases their acceptance and thus, the likelihood of a sustained implementation, as they cite a lack of empirical evidence and usefulness as the main reasons for not implementing/maintaining an approach [6, 22, 160].

Basically, the acceptance of blood collection among athletes is limited. It is thus recommended that practitioners increase athletes’ awareness of innovative approaches, i.e., explaining the rationale for implementation and highlighting potential positive aspects such as injury prevention or performance maximization to improve athlete adherence [16, 17]. Hereby, an interdisciplinary team of physicians, biologists, sports scientists, practitioners, and data analysts for blood collection, analysis, and interpretation generates added value. In particular, the data aspect has gained importance owing to the knowledge of highly individual levels and also because of the large amount of data being compared as the typical reference ranges do not apply here. While physicians are familiar with recognizing abnormal changes in health screening variables, interpreting them, and intervening appropriately [27], data analysts could help analyze, visualize, and interpret data using appropriate basic and innovative powerful statistical methods.

Practical implications with respect to preliminary considerations:

- Is the reason for blood sampling defined?
- Is a team available for blood collection, data analysis, and interpretation?
- What about coach/staff and athlete buy-in?

### Marker Selection and Preanalytical Conditions

As mentioned above, the criteria that an appropriate biomarker should meet include that it is specific/responsive to load, that it does not interfere with training, that it can be measured frequently, that it is robust against confounding variables, and that it provides a rapid result without delay [9, 12]. In practice, this often translates into the use of routine parameters, as described in Sect. 3. However, as the example of CK illustrates, several confounding factors can significantly affect the outcome, which can make load management vulnerable to poor decisions. Although novel innovative biomarkers are increasingly researched in sports, they are far from routine application. The quantification of a load-sensitive cytokine profile, for instance, is usually carried out using the time-consuming enzyme-linked immunosorbent assay method, which leads to a retrospective view of the exercise and its effects on biomarker levels, limiting the suitability [27]. However, as technology is constantly evolving, some promising biomarkers may become rapidly measurable in the near future.

Preanalytical conditions could be a major source of measurement error. It is recommended to avoid vigorous exercise the day before collection, fast overnight, adhere to laboratory guidelines [27], and to choose a standardized time of day to account for diurnal variations. However, some requirements, such as refraining from intense exercise before blood sampling or consistently choosing the same time points, are difficult to fulfill in elite sports settings; a situation that Buchheit recently called “ideal vs real” [161]. Elite athletes exercise on a daily basis and blood collection must be integrated into this training regimen in a time-saving manner. Furthermore, it makes little sense not to train vigorously, as practitioners seek to monitor these very training sessions and their effects. Therefore, it is likely that the conditions under which the blood collection is performed are subject to variation. The resulting confounding variables (e.g., diurnal variations, nutritional aspects such as fasted vs non-fasted) must be carefully documented to allow any interpretation at all if the chosen markers are not robust against these variables. Unknown or unreported confounding variables may contribute to the variability in levels that may be more or less related to the actual training process.

Practical implications with respect to marker selection and preanalytical conditions:

- Are context-specific and suitable markers selected?
- Is it possible to keep the pre-analytical conditions constant?

### Identifying Meaningful Level Changes

Intra- and inter-individual variance (including unexplained variance) is commonly a hallmark of biomarkers that makes accurate interpretation of levels challenging [39]. In addition, there may be athletes who do not adequately respond with respect to biomarker levels despite experiencing a similar objective load compared to their athlete peers [162]. To enable evidence-based decision making, statistical considerations must be made to distinguish changes in levels from possible measurement errors. But when is a change observed considered a “meaningful” change? Here, within-subject variation is the most critical measure for practitioners in monitoring the individual athlete [163]. The approach of practitioners to identify relevant changes based on their experience with the athlete to make informed decisions [164] appears invalid because of intra- and inter-individual variance and multiple confounding factors that could influence biomarker levels (see Sect. 4). Accordingly, evidence-based decision making requires scientific approaches that include estimates of the measurement’s typical error (TE, i.e., standard deviation in repeated testing, when the true value is stable [163, 164]), individual reference ranges, and a declaration of the minor change worth detecting if these changes are thought to reflect training processes. Typical error determination can be accomplished either by performing a test–retest on a cohort (e.g., the entire team) or by performing a reasonable sample size of replicates of the same test with the same individual but ensuring stable “true values”. If there is no opportunity to perform such testing, the TE can be estimated from the published literature [164], especially for biomarkers using the similar measurement method. However, particularly in the case of biomarkers, it must also be noted that TE does not necessarily behave uniformly between individuals (i.e., the TE can be too small for some individuals and too large for others). This may be especially true for individuals who typically reach high levels. Creating and analyzing subgroups can be a viable solution in these cases [165].

Subsequently, it is important to learn more about the individual athlete through repeated testing under controlled conditions to compensate for measurement error (i.e., measurement noise and biological noise [164, 166]). Different time points before the season are well suited to obtain levels without the influence of a chronic training load and at the same time to accurately characterize athletes. Lee et al. [121] suggested additional time points after the pre-season period, during challenging training weeks, and after serious events such as an injury to obtain holistic information and individual reference ranges. Of note, calculating these ranges is challenging because of the need for an adequate sample size and accurate documentation of possible influencing factors.

Finally, the identification of meaningful changes is daunting, and several concepts exist but to our knowledge, there is no consensus on them. No meaningful change should be assumed if the TE is higher than the observed change. Approaches such as changes exceeding several (e.g., two) times the TE can help to identify relevant and/or systematic changes [163]. To illustrate: if the TE of a CK measurement for the sum of ten measurements is 25 U·L^−1^, an observed change of at least 50 U·L^−1^ would indicate a change rather than a measurement error (this example and its calculation are shown in Table 2). The magnitude of this “signal” compared to its “noise” can reveal more information about the potential meaningfulness. Here, the practitioner must determine the smallest meaningful, or clinically relevant change for a given marker [167]. Following Buchheit and his approach on HR measurement, the definition of clinically relevant changes depends on the training context, the type of adaptations to be monitored, and the variable itself, and should be applied in an individualized manner [161]. However, this will only become feasible if an individual range is known through repeated measurements in individuals.

**Table 2:** Simplified concept for calculating the TE using test and retest in a group setting (based on [163, 164, 166]). TE is calculated by the formula SD/√2

Athlete	CK level of the first measurement 1 (U·L^−1^)	CK level of the second measurement (U·L^−1^)	Difference between measurements
1	135	158	− 23
2	209	240	− 31
3	89	123	− 34
4	311	279	32
5	221	185	36
Mean	193	197	− 4
SD			35
TE			25

*CK* creatine kinase, *SD* standard deviation, *TE* typical error, *U·L−1* units per liter

In this respect, another well-known concept is the Athlete Biological Passport of the World Anti-Doping Agency [168], which may serve as a “flagging system” (i.e., a warning appears if the values are beyond an expected range). While the Athlete Biological Passport should indirectly reveal the effects of prohibited substances through repeated longitudinal measurements of selected blood parameters, a similar load monitoring tool could reveal information about fatigue or recovery as an effect of a training stimulus. Hecksteden and colleagues [25] transferred this concept to biomarker monitoring in sports to create individualized reference ranges with a Bayesian approach using CK and urea as an example. However, this requires that a prior distribution from a population is available, serving to subsequently compute reference ranges for the individual observations that are dynamically adjusted depending on the individual measurements over time. Following the principles of the Athlete Biological Passport (APB), it would be conceivable to create different “modules” related to the above classification in Fig. 1, for example, health monitoring at rest and load monitoring using appropriate parameters.

Other concepts to identify changes exist; however, it is beyond the scope of this article to go into these approaches in detail. In either case, it is helpful that data analysts have a clear idea of how to process and interpret a large amount of data. While these approaches may serve to individualize biomarker data, they do not solve the principal question of finding the minimal change that allows conclusions to be drawn regarding load and recovery management. While for instance, plain confidence interval corridors may serve in the case of an ABP application to detect doping with a certain validity and positive predictive value, the meaning of a sports-related biomarker that leaves such individualized reference corridors still warrants further critical investigation.

Practical implications with respect to detecting changes:

- Is there an approach to detect changes compared to measurement error?
- Is there a concept for the detection of clinically relevant changes?

### Meaningful Changes and the Implications for Load Management

Once a meaningful change is detected, one may assume to adjust the training load. However, in the opinion of our author group, the biomarkers established so far (Sect. 3) should be considered primarily as a helpful support for load management and should not be used as the only basis for decision making because of their limited validity and specificity, high variability, and problems in interpreting levels. Even when incorporating mathematical models such as those presented in Sect. 5.3 (e.g., the Athlete Biological Passport), there is a risk of “false positives” for example, the athlete is misclassified being fatigued because biomarker levels were altered by factors unrelated to training. As mentioned earlier, exceeding a predefined acceptable range also need not be considered per se as a sign of overload, which automatically entails a reduction in the training load.

**Fig. 2:**
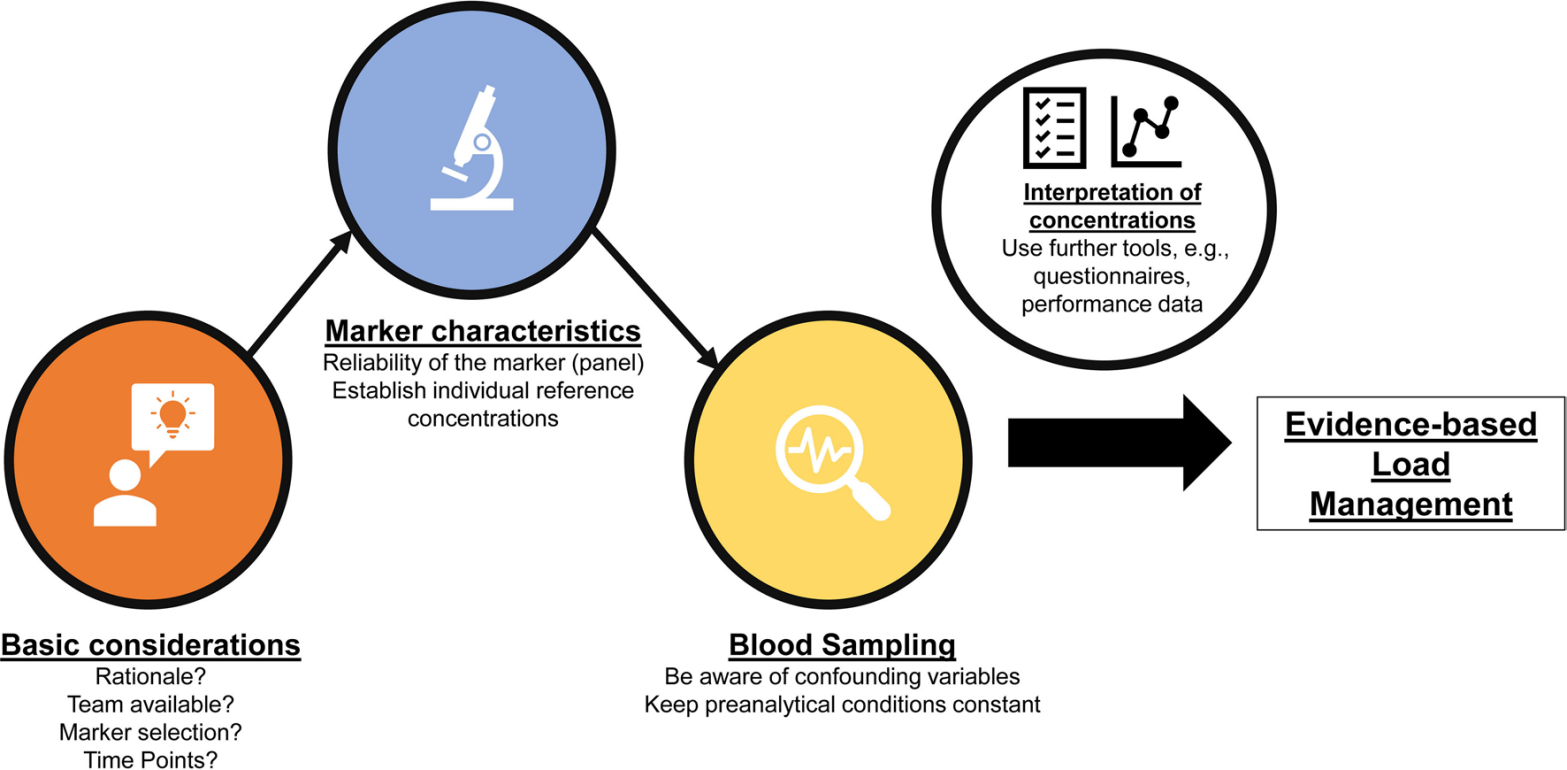
Biomarkers in the context of load management. Following basic considerations, the properties of a marker should be assessed, for example, reliability. Blood collection should be performed with consideration of pre-analytical conditions and confounding variables. Finally, interpretation can be conducted with the help of further monitoring instruments

Not all biomarkers respond reliably to changes in the training load in every athlete and biomarker levels and subjective well-being are generally not well associated. In contrast, questionnaires are normally sensitive to changes in load [39]. Therefore, biomarkers (and other objective measures such as HR variables) should be collected in combination with subjective questionnaires (e.g., RPE, sleep quality). Changes in biomarker levels should also be related to the individual external load and player characteristics (such as age, previous injury, or performance capacity) to create a meaningful context to finally adjust the training load [16, 17, 169, 170]. Because a single biomarker is often not specific to the training load, and misclassifications may occur, further biomarkers covering the same physiological domain [121], and other tools such as questionnaires (e.g., the Profile of Mood States, the Recovery Stress Questionnaire for Athletes, or the Daily Analyses of Life Demands of Athletes [39]) can provide guidance to explain changes in a particular biomarker (e.g., is the CK activity associated with a delayed onset of muscle soreness questionnaire? Do multiple biomarkers of muscle damage increase concurrently?). If different monitoring tools, i.e., objective performance, biomarker levels, and subjective well-being, follow a consistent pattern, this may serve as a solid basis for managing the upcoming loads (Fig. 2).

Finally, resting conditions are often chosen for monitoring purposes, but under these conditions, only small changes in levels may occur “so even an inactive control treatment will appear to produce positive and negative responses” [166]. To avoid the issue of only small increases and their difficult interpretation, a promising option is to monitor changes in biomarkers pre- to post-exercise and to focus on the acute exercise response. In line with this notion, the results of a recent systematic review on hormonal responses to overreaching and overtraining emphasized that resting levels of hormones were generally within the expected ranges. In contrast, growth hormone or adrenocorticotropic hormone was blunted during maximal exercise [171], indicating the potential for post-exercise biomarker sampling.

Practical implications with respect to load management:

- Is there an awareness that adjusting training load based on biomarker changes alone may be inaccurate?
- Are biomarker data also related to the athlete characteristics, performance, and subjective feedback?

### Further Considerations

Despite numerous observational studies (changes in biomarkers in response to exercise), to the best of our knowledge, there are no large cohort studies of athletes in which exercise was controlled/adjusted by changes in biomarker levels or in which biomarkers reliably predicted an outcome such as an injury (in line with [121]). A few pilot studies have been conducted (see Sect. 4), but most of these have involved small sample sizes and/or no control group.

It is further important to note that one marker in principle reflects a limited physiological domain, such as tissue damage. Depending on the sport, there is, however, a complex pattern of physiological responses that are not limited to tissue damage. A one-size-fits-all approach with a single biomarker is therefore rather unrealistic. In addition, considerable intra- and inter-individual variance, responding and non-responding athletes, implies that more than one marker should be included in decision making. A panel that includes aspects of for example, inflammation, muscle status, and injury risk [121] will allow for a more comprehensive picture of athletes. However, the “appropriate” set of markers is currently unknown and it is uncertain how these data should be combined and ultimately interpreted; an issue that could be tackled with novel machine learning approaches capable of processing such a large amount of data. Furthermore, to our knowledge, there are still no reliable and valid markers for aspects such as stress or mental fatigue, which nevertheless influence performance. Therefore, some aspects need to be captured by alternative instruments such as performance tests or questionnaires.

## Conclusion and Outlook

Biomarkers, on the one hand, have the potential to reflect load, fatigue, and recovery processes, and currently used practical markers show certain sensitivity and a partial relationship with the training load. On the other hand, an often unknown physiological background, or a limited analytical and/or clinical validity regarding the load, performance changes, or injury risk emphasizes the need to interpret levels with some caution. Practitioners need to be aware of these pitfalls to avoid misinterpretation of level changes. To this end, the established biomarkers could be used as a helpful adjunct to other tools. External load data and subjective questionnaires could help interpret the biomarker data in an individual and larger context, ultimately leading to evidence-based load management approaches. This requires an interdisciplinary team and ties up corresponding resources. Inconsistent and irregular measurement of certain biomarkers and interpretation without further information seems challenging and should rather be avoided. Single markers and standard statistical methods can give a first insight into internal load and recovery processes. However, multi-marker panels covering load, recovery, and further aspects such as nutrition can provide a deeper insight into an athlete’s physiological processes. Sophisticated methods, such as omics approaches with an advanced data analysis, are on the rise and will be discussed on another occasion.
